# Durable disease control after multimodal therapy and platinum-based chemotherapy rechallenge in advanced clear cell adenocarcinoma of the urethra with lymph node metastases: a case report

**DOI:** 10.3389/fruro.2026.1865730

**Published:** 2026-06-22

**Authors:** Akihiro Maeda, Shohei Tobu, Shuhei Kusano, Maki Kawasaki, Hiroaki Kakinoki, Mitsuru Noguchi

**Affiliations:** Department of Urology, Faculty of Medicine, Saga University, Saga, Japan

**Keywords:** case report, clear cell adenocarcinoma of the urethra, comprehensive genomic profiling, platinum-based chemotherapy, primary urethral carcinoma

## Abstract

**Background:**

Clear cell adenocarcinoma of the urethra (CCAU) is an extremely rare subtype of primary urethral carcinoma. Owing to its low incidence, the optimal treatment strategy for advanced or metastatic disease remains unclear. We report a case of advanced CCAU with lymph node metastases that achieved long-term disease control following multimodal therapy.

**Case presentation:**

A 60-year-old woman presented with urinary retention and was diagnosed with primary urethral clear cell adenocarcinoma (cT4N2M0) with pelvic lymph node metastases. Neoadjuvant gemcitabine plus cisplatin (GC) chemotherapy achieved a partial response and was followed by robot-assisted radical cystourethrectomy combined with hysterectomy, vaginectomy, and pelvic lymph node dissection. Pathological examination revealed ypT4N2M0 disease with negative surgical margins. Adjuvant nivolumab was initiated but was discontinued because of immune-related hypothyroidism and nodal recurrence. Comprehensive genomic profiling demonstrated microsatellite stability, a low tumor mutational burden (1 mutation/Mb), and MET and MYC amplifications. As no actionable alterations were identified, GC chemotherapy was reintroduced and again achieved a partial response. The patient remains progression-free 32 months after diagnosis.

**Conclusions:**

This case suggests that multimodal treatment combining radical surgery and platinum-based chemotherapy may contribute to durable disease control in selected patients with advanced CCAU and lymph node metastasis. Although genomic profiling did not directly guide therapy in this case, molecular characterization may provide a basis for future individualized treatment strategies for this rare malignancy.

## Introduction

Clear cell adenocarcinoma of the urethra (CCAU) is an extremely rare subtype of lower urinary tract malignancy, and most of the relevant literature consists of isolated case reports and literature reviews (1–3). Owing to its rarity, there is no clear consensus regarding its clinical characteristics, prognosis, or optimal treatment strategy. In particular, the management of locally advanced or metastatic disease remains unclear, and available evidence is limited.

We herein report a case of advanced CCAU with lymph node metastases that achieved durable disease control through multimodal treatment, including neoadjuvant chemotherapy, radical surgical resection, and chemotherapy rechallenge after recurrence. We describe the clinical course of this case and discuss the treatment strategy for advanced CCAU in the context of the existing literature.

## Case presentation

A 60-year-old woman with no remarkable past medical history presented to a local hospital with urinary retention. Cystoscopy revealed a nodular tumor compressing the urethral lumen and protruding toward the bladder neck. Contrast-enhanced computed tomography (CT) and magnetic resonance imaging (MRI) demonstrated a urethral tumor invading the bladder and vagina, along with multiple enlarged pelvic lymph nodes (**Figure 1**).

**Figure 1:**
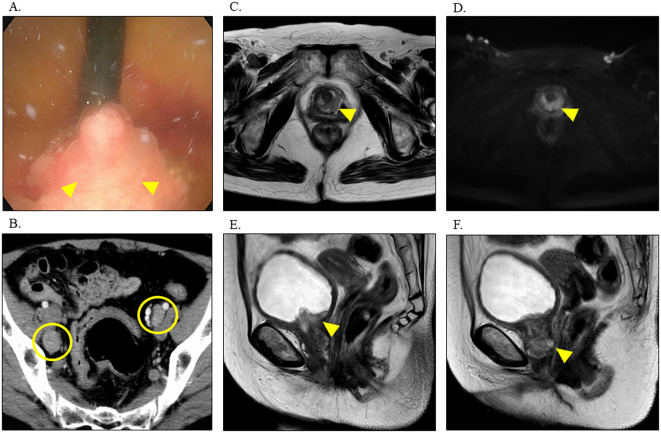
Cystoscopic and radiological findings of the primary urethral tumor. **(A)** Retroflex cystoscopic image showing a urethral tumor extending into the bladder (yellow arrowhead). **(B)** Contrast-enhanced CT showing enlarged pelvic lymph nodes (yellow circles). **(C)** Axial T2-weighted MRI demonstrating a periurethral cystic lesion suggestive of a urethral diverticulum, within which a tumor is identified (yellow arrowhead). **(D)** Diffusion-weighted MRI showing restricted diffusion within the tumor (yellow arrowhead). **(E)** Sagittal T2-weighted MRI showing invasion of the bladder by the tumor (yellow arrowhead). **(F)** Sagittal T2-weighted MRI showing the tumor protruding into the vagina (yellow arrowhead).

A transurethral biopsy of the urethral tumor and a transvaginal biopsy were performed, and histopathological examination revealed clear cell carcinoma. Immunohistochemical staining was positive for CK7, PAX8, AMACR, HNF1α, and p53, and negative for CK20, GATA3, p40, calretinin, ER, and CK10, with a Ki-67 labeling index of 80%. The patient was referred to our department for further evaluation and treatment and was diagnosed with primary CCAU, cT4N2M0, based on imaging and pathological findings.

Because the disease was locally advanced with lymph node metastases, neoadjuvant chemotherapy was administered as the initial treatment. Two cycles of gemcitabine plus cisplatin (GC) chemotherapy were administered, and radiologic response was assessed on serial CT scans according to RECIST version 1.1, based on changes in the size of the metastatic lymph nodes. CT performed 2 months after treatment initiation demonstrated a 37.5% reduction in lymph node size, corresponding to a partial response (PR).

After neoadjuvant chemotherapy, the patient underwent robot-assisted radical cystourethrectomy with hysterectomy, vaginectomy, pelvic lymph node dissection, and bilateral cutaneous ureterostomy. The operative time was 9 h 00 min, including a console time of 4 h 26 min, and the estimated blood loss was 118 mL. No blood transfusion or perioperative complications were observed.

Postoperative pathological examination confirmed primary CCAU with ypT4N2M0 status. Tumor invasion of the bladder, vagina, and uterus was observed; however, the surgical margins were negative. Metastases were identified in nine of the 13 dissected lymph nodes, including the right and left obturator lymph nodes, with maximum diameters of 2.3 cm and 1.5 cm, respectively. Representative histopathological and immunohistochemical findings of the resected urethral tumor are shown in **Figure 2**. Immunohistochemical staining demonstrated positivity for CK7, PAX8, AMACR, HNF1-β, p53, and Napsin A and negativity for CK20, GATA3, ER, WT-1, p63, and CK34βE12. PD-L1 (28-8) and PD-L1 (22C3) staining showed weak membranous positivity in a small subset of tumor cells.

**Figure 2:**
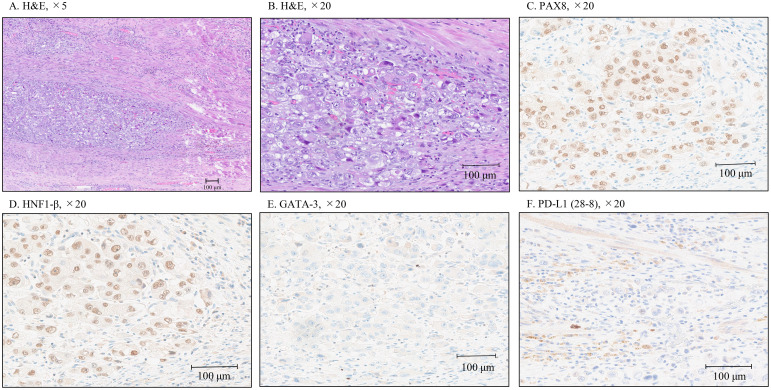
Histopathological and immunohistochemical findings of the resected urethral tumor. **(A)** Low-power H&E image showing infiltrative tumor growth (×5). **(B)** Higher-power H&E image showing tumor cells with clear to eosinophilic cytoplasm, enlarged round nuclei with conspicuous nucleoli, and focal hobnail-like features (×20). **(C)** Immunohistochemical staining for PAX8 showing nuclear positivity in tumor cells (×20). **(D)** Immunohistochemical staining for HNF1-β showing nuclear positivity in tumor cells (×20). **(E)** Immunohistochemical staining for GATA3 showing no significant tumor cell staining (×20). **(F)** PD-L1 (28-8) staining showing weak membranous positivity in a small number of tumor cells (×20).

As postoperative CT showed no apparent residual metastatic lesions, adjuvant nivolumab therapy (240 mg/body every 2 weeks) was initiated. Treatment was started approximately 2 months after surgery, and two cycles were administered. However, approximately 2 months after the initiation of nivolumab, the patient developed immune-related hypothyroidism. In addition, CT performed at the same time demonstrated recurrent pelvic lymph node metastases (**Figure 3A**), and nivolumab was discontinued.

**Figure 3:**
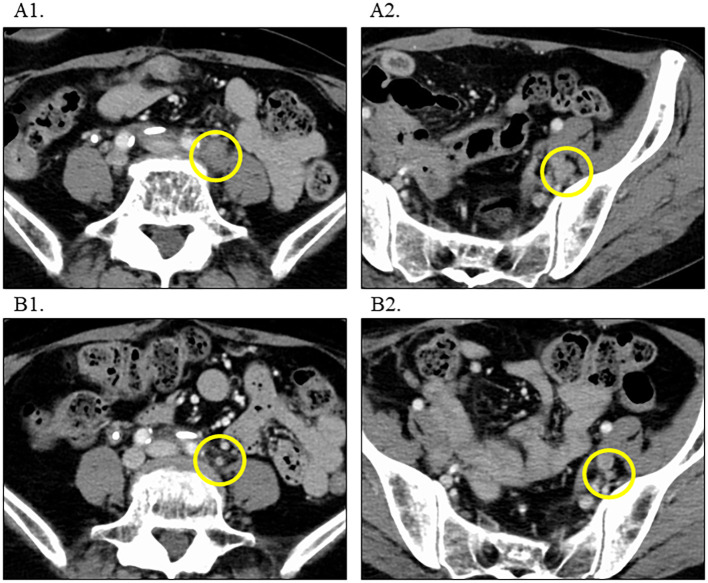
CT findings of recurrent pelvic lymph node metastases before and after re-administration of gemcitabine plus cisplatin (GC) chemotherapy. **(A**1, **B**1**)** show the left common iliac lymph node, and **(A**2, **B**2**)** show the left obturator lymph node. **(A**1, **A**2**)**, CT images obtained at the time of recurrence showing enlargement of the recurrent lymph nodes (yellow circles). **(B**1, **B**2**)**, CT images obtained after two additional cycles of GC chemotherapy showing reduction in lymph node size (yellow circles), corresponding to a partial response.

Approximately 3 months after the initial surgery, comprehensive genomic profiling (FoundationOne CDx) was performed using tumor tissue previously obtained at the referring hospital. The tumor was microsatellite stable (MSS), and the tumor mutational burden (TMB) was low (1 mutation/Mb). MET, MYC, and LYN amplification, and a subclonal SMARCB1 untranslated region *82C>T alteration were detected; however, no companion diagnostic–eligible genomic alterations were identified. Although MET inhibitors have shown efficacy in other malignancies with MET amplification, they are not approved for urethral carcinoma in Japan. Therefore, molecular-targeted therapy was not introduced, and GC chemotherapy was resumed. After two additional cycles of GC chemotherapy, CT performed approximately 2 months after recurrence was confirmed demonstrated a 42% reduction in lymph node size, corresponding to a PR (**Figure 3B**).

GC chemotherapy was subsequently continued with gradual dose reduction because of concern for cumulative hematologic toxicity, and a total of 15 cycles were administered. Ultimately, both gemcitabine and cisplatin were reduced to approximately 40% of the initial doses. Renal function remained preserved throughout treatment, and no hematologic toxicity requiring treatment discontinuation was observed. In addition, no peripheral neuropathy or alopecia was noted. Because cisplatin-associated ototoxicity was also a concern, the patient was referred to the otolaryngology department, where serial audiometric examinations confirmed the absence of hearing impairment during follow-up. At present, the patient remains alive without disease progression 32 months after diagnosis, 29 months after radical surgery, and 25 months after confirmation of recurrence. The overall clinical course is summarized schematically in **Figure 4**.

**Figure 4:**
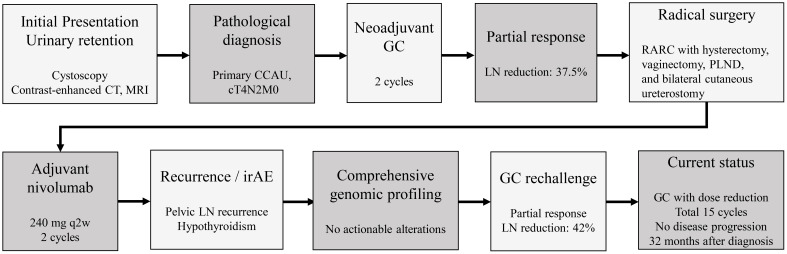
Schematic timeline of the clinical course. Clinical timeline from initial presentation to the most recent follow-up. GC, gemcitabine plus cisplatin; RARC, robot-assisted radical cystourethrectomy; PLND, pelvic lymph node dissection; irAE, immune-related adverse event; LN, lymph node.

## Discussion

CCAU is an extremely rare histological subtype of primary urethral carcinoma. Primary urethral carcinoma is a rare malignancy, accounting for <1% of all genitourinary cancers, and most cases are urothelial carcinoma (4). Adenocarcinoma accounts for approximately 10%–16% of urethral carcinomas, and the clear cell subtype represents only a small fraction of these cases (4–10). In a single-institution series of female urethral carcinomas, clear cell carcinoma accounted for 17% (5/29) of all cases (6). CCAU occurs predominantly in women, and Aron et al. reported that it tends to arise in the proximal urethra and occur in older patients (6). Consequently, evidence for this disease remains limited.

Regarding histogenesis, CCAU is frequently associated with urethral diverticula, and chronic inflammatory changes within a diverticulum have been proposed as a possible mechanism of tumorigenesis (11, 12). However, cases without an associated diverticulum have also been reported, raising the possibility of a periurethral gland origin (9). In the present case, MRI demonstrated a T2-hyperintense periurethral lesion with an intralesional mass showing diffusion restriction, suggesting that the tumor most likely arose within a urethral diverticulum. In the 2022 World Health Organization (WHO) classification, CCAU is categorized as a Müllerian-type tumor based on immunophenotypic features such as PAX8 positivity and GATA3 negativity (10, 13), although a urothelial origin has also been proposed (14, 15).

The present case is notable in that long-term disease control was achieved by stepwise multimodal treatment, including neoadjuvant chemotherapy, radical surgery, adjuvant immunotherapy, and chemotherapy rechallenge after recurrence, in a patient with advanced CCAU with lymph node metastases. CCAU is generally associated with a poor prognosis in locally advanced or node-positive disease. In a review of 33 cases by Jiang et al., the median progression-free survival and overall survival of metastatic cases were reported to be 12 and 28 months, respectively (3). In contrast, our patient has remained alive for 32 months since diagnosis, representing a relatively prolonged clinical course compared with previously reported metastatic cases. Notably, unlike the metastatic cases described previously, our patient achieved durable disease control after GC rechallenge following recurrence, suggesting retained sensitivity to platinum-based chemotherapy even in advanced CCAU. Although no standardized treatment strategy has been established for CCAU, this case underscores the potential value of combining systemic treatment with surgery rather than relying on surgery alone.

Regarding chemotherapy, cisplatin-based regimens have occasionally been reported in individual cases; however, no consensus regimen has been established (3, 4). In the present case, neoadjuvant GC chemotherapy was administered according to treatment strategies used for urothelial carcinoma, resulting in a partial response. Importantly, the re-administration of GC after recurrence also resulted in renewed tumor shrinkage. This clinical course suggests that sensitivity to platinum-based chemotherapy may be retained in CCAU. Although GC rechallenge was continued for a total of 15 cycles in this case, treatment remained feasible with gradual dose reduction to approximately 40% of the initial doses and close monitoring of hematologic toxicity, renal function, neurological symptoms, and hearing function. However, this favorable tolerability should be interpreted cautiously because it is derived from a single patient.

Adjuvant therapy with nivolumab, an immune checkpoint inhibitor, did not produce a clear clinical response in this case. PD-L1 (28-8) staining, which is used as a companion diagnostic assay for nivolumab (16), showed mild membranous positivity in a small subset of tumor cells. Because PD-L1 expression has been associated with response to nivolumab in non-small cell lung cancer (17) and nivolumab has also shown efficacy as adjuvant therapy for urothelial carcinoma (18, 19), adjuvant nivolumab was selected on the basis of extrapolation from postoperative treatment strategies for urothelial carcinoma and the presence of weak membranous PD-L1 positivity in this case. However, no established evidence currently supports the use of immune checkpoint inhibitors in CCAU, and the use of nivolumab in this case should therefore be regarded as exploratory rather than evidence-based for this histological subtype. No obvious clinical response was observed. Subsequent comprehensive genomic profiling revealed that the tumor was microsatellite stable and had a low tumor mutational burden, findings consistent with a tumor biological background less likely to respond to immunotherapy.

In addition, MET and MYC amplifications were detected in this case. Although MET amplification has therapeutic relevance in several other malignancies (20–24), its clinical significance in urethral clear cell adenocarcinoma remains uncertain. At present, the lack of targeted therapy use in this case should be understood not only in terms of regulatory limitations but also in light of the absence of established biological or clinical evidence supporting MET-targeted therapy in CCAU. Therefore, the genomic findings in this case should be interpreted as exploratory and hypothesis-generating rather than directly practice-changing. Previously reported genomic alterations in CCAU include ARID1A, KRAS, and PIK3R1 abnormalities, as well as related signaling pathway alterations (2, 7); however, evidence directly linking these findings to treatment selection remains limited. Nevertheless, molecular profiling data from rare cancers such as CCAU may contribute to future therapeutic investigation.

The tumor in the present case originated from the proximal urethra and extended into the vagina, indicating locally advanced disease. Because the female urethra lies in close proximity to the anterior vaginal wall, tumors extending beyond the periurethral muscle may readily invade the vaginal adventitia and surrounding fibrovascular tissue (6). Therefore, proximal urethral tumors carry a high risk of local extension, and achieving negative surgical margins is critical for local control. This is particularly important because the European Association of Urology guidelines identify high tumor stage, proximal urethral location, and lymph node metastasis as adverse prognostic factors in primary urethral carcinoma (4). In the present case, tumor shrinkage was achieved with neoadjuvant chemotherapy, followed by extensive surgical resection with negative margins, which may have contributed to local disease control. Although radiotherapy alone or chemoradiotherapy has also been reported as a treatment option for female urethral carcinoma (3, 4), local radiotherapy was not selected in this case because the treatment strategy was based on radical surgical resection, and recurrence occurred as systemic disease with multiple lymph node metastases rather than as an isolated local lesion.

This case represents advanced CCAU with lymph node metastases that achieved long-term disease control through multimodal treatment. In this patient, tumor shrinkage was achieved with neoadjuvant GC chemotherapy, followed by radical surgical resection, and renewed tumor shrinkage was obtained by re-administration of GC after recurrence, resulting in durable disease control. These findings raise the possibility that sensitivity to platinum-based chemotherapy may be retained in some cases of advanced CCAU; however, this interpretation is based on a single patient and should be considered hypothesis-generating. Notably, our patient achieved durable disease control after GC rechallenge following recurrence, unlike previously reported metastatic cases. Further accumulation of cases is needed to clarify the optimal systemic treatment strategy for advanced CCAU.

## Conclusion

We report a case of advanced primary clear cell adenocarcinoma of the urethra with lymph node metastases, in which long-term disease control was achieved through multimodal treatment consisting of neoadjuvant chemotherapy, radical surgical resection, and chemotherapy rechallenge after recurrence. Given the rarity of CCAU, no standard treatment strategy has been established. Although the findings from this single case should be interpreted cautiously, they raise the possibility that a treatment approach combining radical surgery with platinum-based chemotherapy may be useful in selected patients with advanced disease. In addition, molecular profiling may provide a basis for future investigation of individualized treatment strategies in this rare malignancy.

## Data Availability

The original contributions presented in the study are included in the article/**Supplementary Material**. Further inquiries can be directed to the corresponding author.
